# BRD4 and Cancer: going beyond transcriptional regulation

**DOI:** 10.1186/s12943-018-0915-9

**Published:** 2018-11-22

**Authors:** Benedetta Donati, Eugenia Lorenzini, Alessia Ciarrocchi

**Affiliations:** Laboratory of Translational Research, Azienda Unità Sanitaria Locale-IRCCS di Reggio Emilia, Viale Risorgimento 80, 42123 Reggio Emilia, Italy

**Keywords:** BRD4, BET inhibitors, Transcriptional regulation, DNA damage response, Telomere regulation, Unconventional function, Cancer

## Abstract

BRD4, member of the Bromodomain and Extraterminal (BET) protein family, is largely acknowledged in cancer for its role in super-enhancers (SEs) organization and oncogenes expression regulation. Inhibition of BRD4 shortcuts the communication between SEs and target promoters with a subsequent cell-specific repression of oncogenes to which cancer cells are addicted and cell death. To date, this is the most credited mechanism of action of BET inhibitors, a class of small molecules targeting BET proteins which are currently in clinical trials in several cancer settings.

However, recent evidence indicates that BRD4 relevance in cancer goes beyond its role in transcription regulation and identifies this protein as a keeper of genome stability.

Indeed, a non-transcriptional role of BRD4 in controlling DNA damage checkpoint activation and repair as well as telomere maintenance has been proposed, throwing new lights into the multiple functions of this protein and opening new perspectives on the use of BETi in cancer. Here we discuss the current available information on non-canonical, non-transcriptional functions of BRD4 and on their implications in cancer biology. Integrating this information with the already known BRD4 role in gene expression regulation, we propose a “common” model to explain BRD4 genomic function. Furthermore, in light of the transversal function of BRD4, we provide new interpretation for the cytotoxic activity of BETi and we discuss new possibilities for a wide and focused employment of these drugs in clinical settings.

## Introduction

BRD4 is a transcriptional and epigenetic regulator that plays a pivotal role during embryogenesis and cancer development. As the other members of the Bromodomains and Extraterminal (BET) family (BRD2, BRD3 and the testis-ovary specific BRDT), BRD4 is characterized by two tandem bromodomains (BD1, BD2). BDs bind acetylated lysine residues on target proteins, including histones [1–3]. Being their affinity higher for proteins with multiple acetylated residues, BRD4 and the other BET proteins, interact with hyper-acetylated histone regions along the chromatin, accumulating on transcriptionally active regulatory elements and promoting gene transcription both at initiation and elongation step [4–6].

Genome wide studies indicate that BRD4 is widely distributed along the genome. However, cancer associated genes seem to be selectively dependent on BRD4 being c-MYC the paradigm of this model [7]. For this reason, inhibition of BRD4 by the use of the recently developed BET-inhibitors (BETi), is currently regarded as one of the most promising strategy to target both hematological and solid malignancies. These molecules mimic the acetyl moiety, occluding the acetyl-lysine binding pocket unique of the BET family proteins, displacing them from chromatin [8–12]. Recent evidence adds further complexity on the role of BRD4 in cancer, showing that this protein plays additional non-transcriptional functions, affecting processes like DNA damage repair and checkpoint activation or telomere homeostasis. BETi mediated inhibition of these BRD4 non-canonical activities could significantly impact on cancer cells growth and survival. In the present work, we attempt to discuss the most recent evidence of a transversal function of BRD4 in keeping genome stability providing new insights into the cytotoxic effects of BETi in cancer cells.

### BRD4 and transcription regulation

**Fig. 1:**
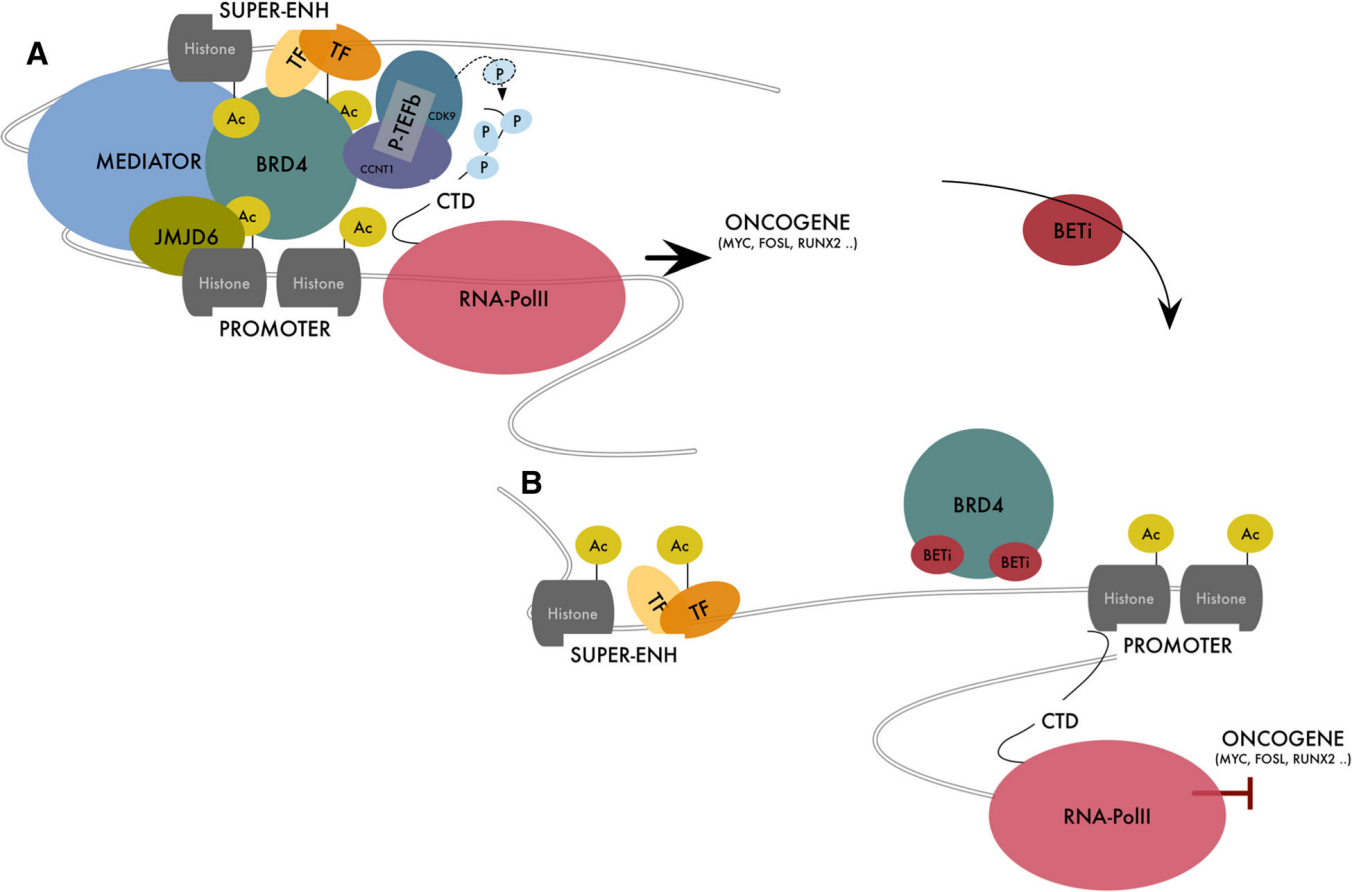
**a** Schematic representation of BRD4 function in the organization and assembly of SE. Binding to hyperacetylated chromatin regions, BRD4 recruits the Mediator complex promoting the assembly of a large platform of transcription regulating proteins, that forms a bridge between SE and Promoter, favoring and stabilizing the binding of RNA-PolII. BRD4 also interacts and activates P-TEFb stimulating transition of RNA-PolII into active elongation. **b** Effect BETi on SE organization. BETi compete with acetylated residues for the binding at the BRD4 bromodomains releasing BRD4 from chromatin and disassembling the interaction between SE and promoter, reducing RNA-PolII throughput and blocking transcription of key oncogenes

BRD4 was first identified as a cell cycle controlling protein, which associates with chromosomes during mitosis to mark genes whose ready transcription in G1 is required to ensure cell cycle progression [13–16]. From these original observations the relevance and the complexity of BRD4 in transcription regulation has grown exponentially (Table 1). The transcriptional activity of BRD4 is essential during embryogenesis [17–19] and for cell identity determination. In the early phases of embryogenesis, BRD4 is required to maintain Embryonic Stem Cells (ESCs) self-renewal and pluripotency by controlling or cooperating with ESC TFs like Nanog [18] and OCT4 [19]. In mice, Brd4-null embryos die shortly after implantation due to their inability to maintain the inner cell mass, which gives rise to ESCs [20]. Later during development BRD4 is essential for cell identity determination through the selective regulation of lineage-specific genes. Lee and colleagues using two conditional knockout mouse models showed that BRD4 expression is required for adipogenesis and myogenesis [21]. As well, Najafowa et al. using human fetal osteoblasts demonstrated that BRD4 activity perturbation impairs the entire osteoblast differentiation process from the early commitment to the late phases of mineralization and bone formation [22]. All these studies converge on a common model: BRD4 accumulates on a specific subset of lineage specific, context dependent ENHs through the interaction with lineage specific TFs, facilitating the expression of cell-identity genes. BRD4 is a transcription activator, even if scattered evidence indicates that BRD4 may work occasionally as transcriptional repressor [6, 23]. As reader of the histone code, BRD4 accumulates on hyper-acetylated and transcriptionally prone chromatin regions (both promoters and ENHs) working as nucleation center for the assembly of large protein complexes that promote RNA-PolII activity stimulating transcription initiation and elongation (Fig. 1a). This function largely but not entirely relies on BRD4 BDs and on their ability to recognize acetyl-proteins [2, 6, 24].

**Table 1:** Key BRD4 target genes in normal and tumor cells

	Gene function	Model	Reference
Embryonic cells			
Oct4	Embryonic development and stem cells pluripotency	mESC	Di Micco R, et al. Cell Rep 2014 [17]
Xite	Regulator of X- chromosome inactivation	mESC	Di Micco R, et al. Cell Rep 2014 [17]
Tsix	Regulator of X- chromosome inactivation	mESC	Di Micco R, et al. Cell Rep 2014 [17]
Nanog	Embryonic stem cells proliferation and pluripotency	mESC	Wu T, et al. Stem Cell Report 2015 [19]
Sox2	Embryonic development and determination of cells fate	mESC	Wu T, et al. Stem Cell Report 2015 [19]
Osteogenesis			
TNFRSF11B	Regulator of osteoblasts differentiation	hFOB	Najafova Z, et al. Nucleic Acids Res 2017 [22]
RUNX2	Regulator of osteoblastic differentiation and skeletal morphogenesis	hFOB	Najafova Z, et al. Nucleic Acids Res 2017 [22]
ALPL	Regulator of osteoblasts differentiation	hFOB	Najafova Z, et al. Nucleic Acids Res 2017 [22]
Adipogenesis			
Pparg	Regulator of adipocytes differentiation	mESC	Lee JE, et al. Nat Commun 2017 [21]
Cebpa	Regulator of adipocytes differentiation	mESC	Lee JE, et al. Nat Commun 2017 [21]
Fabp4	Regulator of fatty acids uptake and metabolism	mESC	Lee JE, et al. Nat Commun 2017 [21]
Myogenesis			
Myod1	Regulator of muscle cells differentiation	mESC	Lee JE, et al. Nat Commun 2017 [21]
Somatic cells			
AURKB	Mediator of cell cycle progression	Human Foreskin Keratinocytes	You J, et al. Mol Cell Biol 2009 [89]
Ran	Regulator of cell cycle progression	Mouse Fybroblast	Dey A, et al. Mol Biol Cell 2009 [14]
Kif5b	Regulator of cell cycle progression	Mouse Fybroblast	Dey A, et al. Mol Biol Cell 2009 [14]
Tgf1	Regulator of cell cycle progression	Mouse Fybroblast	Dey A, et al. Mol Biol Cell 2009 [14]
Ywhah	Regulator of cell cycle progression	Mouse Fybroblast	Dey A, et al. Mol Biol Cell 2009 [14]
Gnb1	Regulator of cell cycle progression	Mouse Fybroblast	Dey A, et al. Mol Biol Cell 2009 [14]
Rad21	Regulator of cell cycle progression	Mouse Fybroblast	Dey A, et al. Mol Biol Cell 2009 [14]
Ctnnd1	Regulator of cell cycle progression	Mouse Fybroblast	Dey A, et al. Mol Biol Cell 2009 [14]
Topo1	Regulator of cell cycle progression	Mouse Fybroblast	Dey A, et al. Mol Biol Cell 2009 [14]
Neuron cells			
Fos	Regulation of synaptic plasticity in neurons	Mouse Neurons	Korb E, et al. Nat Neurosci 2015 [90]
Arc	Regulation of synaptic plasticity in neurons	Mouse Neurons	Korb E, et al. Nat Neurosci 2015 [90]
Nr4a1	Regulation of synaptic plasticity in neurons	Mouse Neurons	Korb E, et al. Nat Neurosci 2015 [90]
Cardiac cells			
Nppa	Cardiovascular homeostasis	Rat cardiac Myocytes	Stratton MS, et al. Cell Rep 2016 [91]
Nppb	Cardiovascular homeostasis	Rat cardiac Myocytes	Stratton MS, et al. Cell Rep 2016 [91]
Ctgf	Chondrocyte proliferation and differentiation	Rat cardiac Myocytes	Stratton MS, et al. Cell Rep 2016 [91]
Pln	Cardiovascular homeostasis	Rat cardiac Myocytes	Stratton MS, et al. Cell Rep 2016 [91]
Myh6/7	Components of cardiac muscle	Rat cardiac Myocytes	Stratton MS, et al. Cell Rep 2016 [91]
Cancer cells			
C-MYC	Proto-oncogene	Multiple Myeloma	Lovén J, et al. Cell 2013 [7]
IGLL5	Regulator of immune response	Multiple Myeloma	Lovén J, et al. Cell 2013 [7]
IRF4	Interferon regulatory factor	Multiple Myeloma	Lovén J, et al. Cell 2013 [7]
XBP1	Post-translational Regulation	Multiple Myeloma	Lovén J, et al. Cell 2013 [7]
PRDM1	Regulator of immune response	Multiple Myeloma	Lovén J, et al. Cell 2013 [7]
PIM2	Proto-oncogene	Retinoblastoma	Rahman S, et al. Mol Cell Biol 2011 [37]
CCND1	Regulator of cell cycle progression	Retinoblastoma	Rahman S, et al. Mol Cell Biol 2011 [37]
DCPS	Regulator of mRNA processing	Retinoblastoma	Rahman S, et al. Mol Cell Biol 2011 [37]
TERT	Regulator of Telomere Homeostasis	Colon Cancer	Akincilar SC, et al. Cancer Discov 2016 [80]
GABPA	Regulator of Mitochondrial function	Colon Cancer	Akincilar SC, et al. Cancer Discov 2016 [80]
KRAS	Proto-oncogene	Glioblastoma	Du Z, et al. Int J Oncol 2018 [92]
BRAF	Proto-oncogene	Glioblastoma	Du Z, et al. Int J Oncol 2018 [92]
CALM2	Regulator of cell cycle progression and proliferation	Glioblastoma	Du Z, et al. Int J Oncol 2018 [92]
ARAF	Proto-oncogene	Glioblastoma	Du Z, et al. Int J Oncol 2018 [92]
MAPK8	Regulator of proliferation,differentiation, transcription and development	Glioblastoma	Du Z, et al. Int J Oncol 2018 [92]
PLCB3	Regulator of signal transduction	Glioblastoma	Du Z, et al. Int J Oncol 2018 [92]
MAPK10	Regulator of proliferation,differentiation, transcription and development	Glioblastoma	Du Z, et al. Int J Oncol 2018 [92]
ADCY6	Regulator of signal transduction	Glioblastoma	Du Z,et al. Int J Oncol 2018 [92]
RUNX2	Regulator of cell proliferation, survival and invasiveness	Thyroid, Breast Cancer	Sancisi V, et al. Nucleic Acids Res 2017 [11]
FOSL1	Regulator of cell proliferation, differentiation and trasformation	Non Small Cell Lung Cancer	Shi J, et al. Molecular Cell 2014 [6]
BCL2	Regulator of apoptosis	Hematological malignancies	Shi J, et al. Molecular Cell 2014 [6]
XRCC5	Involved in DSB DNA repair	Prostate Cancer	Li XY, et al. Cell Rep 2018 [58]
XRCC4	Involved in DSB DNA repair	Prostate Cancer	Li XY, et al. Cell Rep 2018 [58]
NHEJ1	Involved in DSB DNA repair	Prostate Cancer	Li XY, et al. Cell Rep 2018 [58]
WRN	Involved in DNA repair and maintenance of genome stability	Prostate Cancer	Li XY, et al. Cell Rep 2018 [58]
DCLRE1C	Involved in DSB DNA repair	Prostate Cancer	Li XY, et al. Cell Rep 2018 [58]
LIG4	Involved in DSB DNA repair	Prostate Cancer	Li XY, et al. Cell Rep 2018 [58]
ERCC4	Involved in DNA repair	Prostate Cancer	Li XY, et al. Cell Rep 2018 [58]
HMOX1	Involved in oxidative stress response	Prostate Cancer	Hussong H, et al. Cell Death Dis 2014 [93]
SESN3	Involved in oxidative stress response	Prostate Cancer	Hussong H, et al. Cell Death Dis 2014 [93]
HDAC6	Hystone Deacetylase	Prostate Cancer	Hussong H, et al. Cell Death Dis 2014 [93]
KEAP1	Involved in oxidative stress response	Prostate Cancer	Hussong H, et al. Cell Death Dis 2014 [93]
MAPK3	Regulator of proliferation, differentiation, transcription and development	Prostate Cancer	Hussong H, et al. Cell Death Dis 2014 [93]
VIM	Involved in maintenance of cell integrity	Prostate Cancer	Hussong H, et al. Cell Death Dis 2014 [93]
NF-kB signaling pathway genes			
TNFα	Proinflammatory cytokine	Non Small Cell Lung Cancer, Akute Myeloid Leukemia	Huang B, et al. Mol Cell Biol 2009 [32]
E-Selectin	Inflammatory response mediator	Non Small Cell Lung Cancer	Huang B, et al. Mol Cell Biol 2009 [32]
IL8	Inflammatory response mediator	THP1	Huang B, et al. Mol Cell Biol 2009 [32]
Estrogen receptor target genes			
GREB1	Involved in response to estrogen signalling	Endometrial and breast cancer	Nagarajan S, et al. Cell Rep 2014 [94]
TFF1	Involved in response to estrogen signalling	Endometrial and breast cancer	Nagarajan S, et al. Cell Rep 2014 [94]
Androgen receptor target genes			
PSA	Biomarker of prostatic carcinoma released in seminal plasma	Prostate Cancer	Urbanucci A, et a Cell Rep 2017 [95]
CAMKK2	Regulator of signal transduction	Prostate Cancer	Urbanucci A, et a Cell Rep 2017 [95]
UBE2C	Involved in protein ubiquitination regulating cell cycle progression	Prostate Cancer	Urbanucci A, et a Cell Rep 2017 [95]
HOXB13	Transcription factor involved in embryonal development	Prostate Cancer	Urbanucci A, et a Cell Rep 2017 [95]
AURKA	Mediator of cell cycle progression	Prostate Cancer	Urbanucci A, et a Cell Rep 2017 [95]

#### BRD4 and transcription initiation

Transcription initiation begins with the recruitment of RNA-PolII on the preinitiation complex (PIC) at the gene promoter, followed by RNA-PolII phosphorylation at Serine 5 and stabilization of the RNA-PolII-promoter interaction. PIC assembly is widely influenced by ENHs and controlled by TFs and other transcription regulatory proteins [25]. Mediator of RNA-PolII transcription (Mediator) is a large complex with modular organization which transduces signals from TFs and activators at the ENHs to promoters, timing PIC formation and transcription initiation [26–28]. BRD4 has been isolated as preferred cofactor of the Mediator complex [29–31]. BRD4 and the Mediator complex have been shown to largely co-localize at active ENHs and in particular at the level of super-ENHs-SE and their genomic localization is disrupted by BET inhibition (Fig. 1a, b). Indeed, early after BETi treatment Mediator detaches by the set of regulatory elements to which is bound and its displacement tracked closely with the sensitivity of gene expression to BETi [7, 29]. Beside highlighting the relevance of BRD4 in mediating the crosstalk between promoters and distal ENHs these data indicate that inhibition of Mediator is one of the central mechanisms underneath BETi cytotoxicity.

Many TFs and chromatin remodeling proteins have been shown to interact with BRD4 [6, 32, 33]. Since TFs are mainly associated with ENHs the interplay with these proteins is crucial for BRD4-ENHs interaction and to define BRD4 selectivity for target genes. TFs recruit acetyltransferases like p300/CBP, which in turn promote nucleosome and/or non histonic proteins (like the same TFs) acetylation. Therefore, recruitment of BRD4 to active ENHs is likely mediated by interaction with acetylated histones as well as direct binding to ENH-associated TFs [6]. Indeed, a recent model proposed that the simultaneous binding of BRD4 to histones and TFs would stabilize TFs binding to ENHs maintaining elevated the transcriptional activity of these elements. The interaction of BRD4 with TFs and histone modifiers can be either mediated by BDs and therefore dependent on the acetylation status of TFs [32, 34, 35] or by other BRD4 domains, thus being not affected by BETi treatment [33, 36, 37]. It has been shown that the ET domain of BRD4 is involved in transcriptional regulation by interacting with histone modifiers including the arginine demethylase JMJD6 [36], the lysine methyltransferase NSD3 and the nucleosome remodeling enzymes SWIF/SNF and CDH4 [37]. Through these interactions BRD4 facilitates transcriptional activation by de-compacting chromatin and accelerating messenger RNA synthesis. Using a yeast purified FLAG-tagged BRD4, Wu and colleagues performed an unbiased biochemical screening looking for BRD4 interactors [33]. Beside identifying consolidated BRD4 partners and many chromatin modifiers, these Authors demonstrated that BRD4 associates in a BD independent mode with a selected group of TFs including p53, YY1, AP2, c-JUN, C/EBPα and β and the heterodimer c-MYC/MAX. BRD4 interaction with p53 is mediated by two regions outside the BDs and in part regulated by casein kinase (CK2) mediated phosphorylation. CK2 phosphorylates BRD4 in an acidic region juxtaposed to BDs enabling BRD4 loading on DNA and interaction with p53. The selective binding with certain TFs as well as the possible modulation of these interactions by signaling pathways, have major implication in dictating BRD4 specificity for its target genes and may strongly contribute to BRD4 context-dependent functions. Indeed, Najafova and colleagues, studying osteoblast differentiation, showed that BRD4 is enriched at putative osteoblast specific distal ENHs where BRD4 colocalizes specifically with a restricted set of TFs including C/EBPb, TEAD1, FOSL2 and JUND [22]. Also in acute myeloid leukemia, BRD4 has been shown to co-localize and cooperate with hematopoietic TFs (including PU.1, FLI1, ERG, C/EBPα, C/EBPβ, and MYB) in conjunction with the lysine acetyltransferase p300/CBP to support lineage-specific transcriptional circuits in this disease [38]. BRD4 has been also shown as necessary coactivator of the inflammatory transcriptional program driven by NF-κB by binding to acetylated RelA [32, 34] and of a diacetylated form of TWIST in triple negative breast cancer. Beside controlling protein coding genes, BRD4 also regulates transcription of ENH-associated RNA (eRNAs) [39]. Many active ENHs have been shown to be site of active transcription for short (less than 200 nt) and bidirectional RNAs, which in turn assist ENHs in promoting target genes expression [40, 41]. Inhibition of BRD4 by BETi treatment has been shown to consistently repress the expression of a pool of eRNAs transcribed from BRD4 enriched ENHs. Treatment with BETi disassociates BRD4 from target ENHs abolishing BRD4-RNAPolII interaction at these sites reducing RNA-PolII recruitment with consequent inhibition of eRNA synthesis [39]. Promoting ENHs active transcription, BRD4 fosters ENHs activity not only through an architectural remodeling.

#### BRD4 and transcription elongation

RNA-PolII pausing at promoter-proximal regions and its release into active transcription are a major rate limiting steps of gene expression [25]. Release of RNA-PolII into active transcription is mediated by the positive transcription elongation factor-b (P-TEFb) complex, comprising cyclin T1 (CCNT1) and cyclin-dependent kinase 9 (CDK9). In its active form, P-TEFb complex interacts with TFs and cofactors, phosphorylates pausing factors (like the Negative Elongation Factor complex NELF) displacing their binding from chromatin, and phosphorylates the carboxy-terminal domain (CTD) of RNA-PolII at Serine 2 activating transcription [25]. Among the 3 ubiquitously expressed BRD proteins, BRD4 is the only one able to interact with p-TEFb through its C-terminal domain [14, 16, 42–45]. Interaction with BRD4 prevents P-TEFb binding to the inhibitory ribonucleoprotein complex 7SK/HEXIM that sequesters P-TEFb in its inactive form [43, 45]. Accumulation of BRD4 on hyper-acetylated and transcriptionally active TSSs serves as docking sites for P-TEFb which in turns facilitates activation of RNA-PolII and its release into active elongation. Recent evidence indicates that also ENH-associated BRD4, through its interaction with JMJD6, influences P-TEFb activity promoting elongation [36]. JMJD6 colocalizes with BRD4 selectively on a definite set of active distal ENHs characterized by high levels of H3K4Me1 and H3K27Ac and named anti-pause ENHs. BRD4 mediates JMJD6 recruitment on these sites that in turns 1. demethylates H4 arginine 3 mono- and dimethylated (H4R3me1, H4R3me2) which are associated with transcription repression, 2. demethylates the 5′-methyl-phosphate cap of 7SK RNA causing its degradation and promoting local activation of P-TEFb. Long range chromatin looping mediates the effect of ENH-associated BRD4/JMJD6/P-TEFb complex on RNA-PolII release at TSS of target genes. Nucleosomes can also impede RNA-PolII progression along the gene body reducing transcript elongation and gene expression [36]. A recent report by Kanno and colleagues showed that BRD4 facilitates RNA-PolII transition along the gene body in a P-TEFb independent manner, further promoting transcript elongation [39]. These Authors showed that BRD4 beside being enriched on active promoters and ENHs is bound along the body of highly transcribed genes. Gene expression alterations following BRD4 inhibition either by BETi or BRD4 silencing largely correlate with BRD4 localization along gene bodies. This indicates that the removal of BRD4 from these genes has deleterious consequences for the RNA-PolII processivity. BRD4 follows RNA-PolII during elongation since abrogation of transcription by Actinomycin-b dramatically reduces the amount of BRD4 bound along target genes. Based on this evidence the Authors proposed that BRD4 functions as chaperone that taking advantage of its ability to interact with acetylated histones facilitates the passage of RNA-PolII through hyperacetylated nucleosomes [39]. Linked to the effect of BRD4 on transcription elongation, Donato et al. recently proposed a compensatory mechanism that may affect BETi gene target selectivity. They indicated that BETi unsensitive genes can compensate the impairment of transcription elongation induced by BRD4 inhibition by increasing RNA-PolII recruitment at TSS and transcription initiation. This is not possible for BRD4-addicted genes which have already maximized the amount of RNA-PolII loaded at TSS, to sustain the high rate of required transcription. The block of transcription elongation imposed by BRD4 inhibition impedes promoter clearance and further recruitment of RNA-PolII conferring selective susceptibility to BETi to highly expressed genes [46].

In addition to function as scaffolding platform for a variety of TFs and transcription related proteins, BRD4 has been shown to directly affect RNA-PolII activity by its intrinsic kinase and acetyl-transferase activity [47–49].

BRD4 directly stimulates RNA-PolII CTD domain phosphorylation at Serine 2 triggering productive transcription [49, 50]. Noticeably, BRD4-mediated RNA-PolII phosphorylation results in the specific activation of Topoisomerase I which associates with RNA-PolII during transcription progression favoring DNA unwinding and RNA-PolII transition [51]. Furthermore, BRD4 has been shown to phosphorylate CDK9 (of the P-TEFb complex) regulating its enzymatic activity. As histone acetyltransferase (HAT) BRD4 has been shown to acetylate histones H3 and H4 with a distinct pattern, different from those of classical HATs. BRD4 acetylates H3 K122 resulting in nucleosome eviction and chromatin deconstruction leading to increased transcription [47].

#### BRD4 and super-ENHs (SEs): Defining the molecular bases of BETi cancer specificity

Considered the centrality and the multifunctional role that BRD4 plays in transcription regulation it is not surprising that BETi represent an attracting strategy in cancer therapy. However, while the successful employment of these drugs is moving faster from selected MYC-addicted hematological malignancies to solid cancers, questions about cancer specific susceptibility to these drugs still remain. Gene expression profiles showed that BRD4 is expressed in a broad range of somatic cells. As well, ChIP seq data showed that BRD4 occupies many ENHs and promoters also in non-cancer cells and generally contributes to gene expression (Table 1) [6]. Thus, how inhibition of a global chromatin regulator as BRD4 may results in a such cancer selective targeting, it remains at least in part unclear. The recent discovery of SEs suggests that BETi cancer specificity may be a matter of quantity [7, 52]. SEs are large clusters of ENHs closely spaced in genome that possess unique functional properties distinguishing them from regular ENHs [52]. These regions show a density of transcription factors and regulators binding as well as a quantity of H3K27Ac and H3K3Me1 that surmount of one order of magnitude the one detected on regular ENHs. In cancer, SEs are central to maintain cell identity and are deputy to drive the expression of specific oncogenes to which cancer cells become highly addicted. Genome-wide studies indicated that SEs mark lineage specific TFs and oncogenes in a wide range of cancer type, revealing that these structures are acquired during tumorigenesis and are unique to cancer cells while largely absent in untrasformed cells [7, 53, 54].

Thus, shortcutting the functional organization of these structures causes a dramatic drop in oncogenes expression and consequential cancer cells growth inhibition or death.

In the attempt of defining the molecular mechanisms beyond the cancer cytotoxicity of BETi in Multiple Myeloma (MM), Loven and colleagues showed that BRD4, together with Mediator, and P-TEFb is heavily accumulated on SEs and that BETi causes a preferential loss of BRD4 at these elements. Inhibition of BRD4 then resulted in loss of SEs function with consequent transcription elongation defects and block of oncogenes expression including c-MYC or other SEs associated MM relevant genes such as IGLL5, IRF4 and XBP1 [7]. At SEs, BRD4, together with Mediator, acts as the catalyst of the assembly of a cooperative and synergistic high density transcriptional complexes changing structure, dynamics and function of chromatin. Sabari and colleagues recently investigated the biophysical nature of SEs showing that these elements exhibit properties of liquid-like condensates. They showed that accumulation of BRD4 and Mediator at SEs forms nuclear puncta and gives rise to phase-separated droplets that compartmentalize and concentrate the transcription apparatus favoring protein crosstalk and interplay [55]. In such environment, inhibition of BRD4 has greater consequences that on regular ENHs conferring to SE-associated genes a higher magnitude of susceptibility to BETi and explaining at least in part the cancer selective activity of these drugs (Fig. 1b).

### BRD4 and DNA damage

Perturbations of DNA double strand breaks (DSBs) repair contributes to genome instability and cancer development [56]. Alterations in the chromatin structure, largely mediated by histone posttranslational modifications, are implicated in the initiation and propagation of DNA damage response [57]. BRD4 reader of the chromatin state and pivot of chromatin organization has recently been proposed as major player in the DNA damage repair and propagation, to which it contributes through both canonical and unconventional mechanisms. Besides being a master regulator of many genes part of the DNA repair system or involved in DNA damage checkpoint activation, BRD4 also contributes to DNA damage repair in a transcriptionally independent manner.

**Fig. 2:**
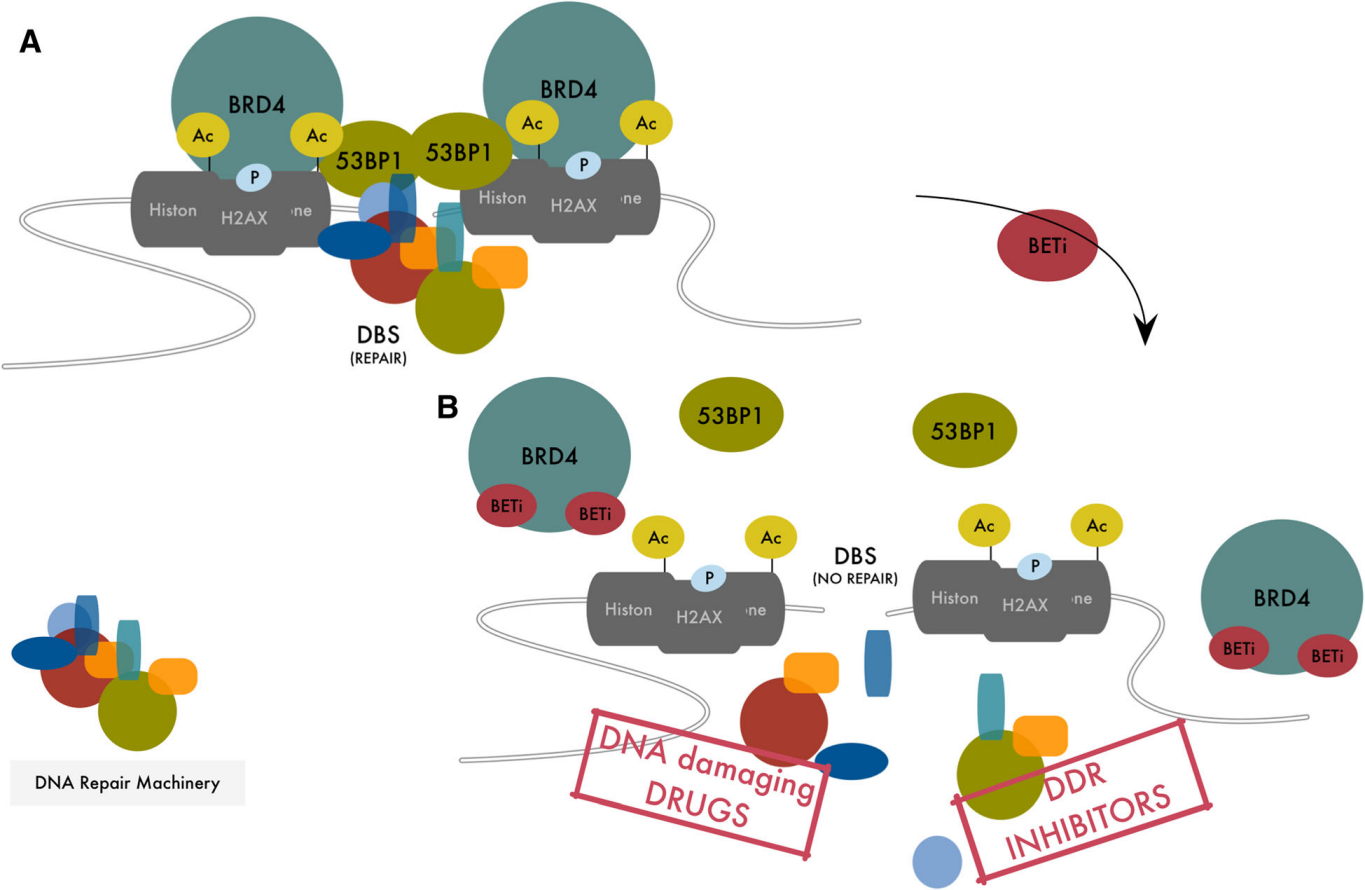
**a** Schematic representation of BRD4 function in DSB repair. H4Ac and γH2AX accumulate at DSBs triggering BRD4 recruitment. BRD4 facilitates and stabilizes the interaction of 53BP1 that in turn serves as adaptor for the assembly and activation of DNA repair machinery. **b** Effect of BETi on DNA repair system. BETi compete with acetylated residues for the binding at the BRD4 bromodomains releasing BRD4 from DSBs, destabilizing the DNA repair machinery and inducing accumulation of DNA alterations up to cell death. The function of BRD4 on DNA repair suggests a possible synergistic effect of BETi and DNA damaging agents (like radiation or platinum-based therapy) or specific inhibitors of DDR

In particular, two distinct papers reported that BRD4 is necessary for the correct activation of the Non Homologus End Joining (NHEJ) recombination pathway [58, 59]. Stanlie et al. reported that in B lymphocyte, BRD4 is required during immunoglobulin (Ig) isotype switching for completion of class switch recombination after DSBs by Activation Induced cytidine Deaminase (AID) [59]. Li et al. also showed that BRD4 is required for repair of DNA DSBs induced by ionizing radiation (IR) and promotes gene rearrangements in prostate cancer cells. In presence of induced DSBs (for example by IR) BRD4 inhibition, either by BETi or specific silencing, resulted in enhanced phosphorylated H2AX (γH2AX) and persistence of DNA damage favoring genomic catastrophe and ultimately cell death [58]. In both works, the effect of BRD4 on DNA repair system, was not dependent on BRD4 transcriptional activity, but on its ability of forming a platform between histone modifications and components of the DNA repair machinery (Fig. 2a).

Mechanistically, DNA DSBs are accompanied by increased H4 acetylation (H4Ac) and phosphorylation of H2AX (γH2AX). Accumulation of these modifications at both ends of the breaks induces BRD4 recruitment that in turn works as docking sites for the DNA repair complex (Fig. 2a).

Among many DNA repair components that show interaction with BRD4 in co-immunoprecipitation experiments, both works identified the p53 Binding Protein (53BP1) as major binding partner of BRD4 in DNA damage repair regulation [58, 59]. 53BP1 is a large protein of 1972 amino acids that has no apparent enzymatic activity but when recruited to DSB specific histone code acts as a molecular scaffold that recruits additional DSB-responsive proteins to damaged chromatin [60, 61].

Using sequential Chromatin Immune Precipitation experiments, Li et al. demonstrated that pharmacological inhibition or silencing of BRD4 abolish 53BP1 recruitment to IR induced DSB. By contrast, silencing of 53BP1 does not affect BRD4 binding in the same condition, demonstrating that hierarchically BRD4 functions upstream of 53BP1 and serves as a bookmark to guide this protein on DSB sites. Even if further evidence is needed, it is likely that the interaction with BRD4 at DSBs serves to stabilize the binding of 53BP1 with DNA repair complexes on site, in a model that tightly resembles the mechanism of action of BRD4 in stabilizing transcriptional machinery at enhancers and promoters during transcription [58] (Fig. 2a-b). Besides, BRD4, relying on its enzymatic activities, could promote acetylation or phosphorylation of DNA repair proteins stimulating their cooperation. This may lead to the formation of dense multi-protein complexes that change the physical status of chromatin (liquid-liquid phase separation) resembling the model already proposed for super-enhancers [55].

In addition to its role in promoting NHEJ activity, BRD4 has been also implicated in the activation of DNA damage checkpoint [62, 63].

Replication stress, induced among the others by oncogene increased transcription or DNA replication, is a complex phenomenon which has serious implications for genome stability [64]. Generation of aberrant replication fork structures exposes single-stranded DNA which in turn activates the replication stress response including the Ataxia Telangiectasia And Rad3-Related (ATR)- Checkpoint kinase 1 (CHK1) kinase axes [65]. Malfunctioning of the DNA damage checkpoint leads indeed to collapse of replication fork, DNA DSB and chromosome rearrangement [66]. Evidence exists that BRD4 associates with chromatin at the level of replication forks. Zhang and colleagues showed that BRD4 interacts with several components of the DNA pre-replication complex and regulates activation of DNA damage response in a transcriptionally independent way. The Cell Division Cycle 6 (CDC6), licensing factor of DNA replication initiation, is crucial for BRD4 function in this process. BRD4 inhibition either by BETi or specific silencing caused defective DNA damage checkpoint sensitizing cells to stress-inducing agents [63].

Partially in contraposition with these data, Floyd and colleagues showed that a specific isoform (iso B) of BRD4 functions as endogenous inhibitor of DNA damage response, insulating chromatin from DNA damage response propagation. In response to induced DNA damage, increased association of BRD4 Iso B with chromatin is observed. In turn, BRD4 isoB recruits Structural Maintenance of Chromosomes 2 and 4 (SMC2, SMC4), components of the condensing II complex inducing chromatin condensation. Inhibition of this BRD4 variant induces relaxed chromatin structure and enhanced survival after irradiation. Of note, Iso B lacks the C-terminal domain through which BRD4 interacts with p-TEF and is necessary for its transcriptional activity [62]. This structural difference may account at least in part for this peculiar function of BRD4.

The functional involvement of BRD4 in DNA damage checkpoint activation and DNA repair holds relevant implications for cancer therapy and envisages new settings for the employment of BETi both alone or in combination.

Targeting DNA damage repair (DDR) pathways is a promising therapeutic strategy in treating cancer with defective DDR components [67]. Indeed, PARP inhibitors have shown solid anti-tumor activity in the treatment of BRCA1 and BRCA2 mutated ovarian and breast cancers [68–70]. As well, ATR and CHK1 inhibitors have shown selective cytotoxic effects on Cyclin E1 (CCNE1) overexpressing cancer due to the high dependency of these tumors on the ATR/CHK1 axis [71]. The evidence that BRD4 positively regulates DNA damage checkpoint activation and DSB repair, implicates that its pharmacological inhibition by BETi may increase sensitivity of cancer cells to DDR or ATR inhibitors defining a possible combination strategy in specific tumor settings. Indeed, a recent report showed that BRD4 inhibition by BETi induces recombination deficiency and sensitize cancer cells to PARP inhibitors regardless of BRCA1/2, p53 and RAS/RAF mutational status. These results not only demonstrate the synergistic effects of BETi in combination with DDR targeting agents but also imply the possibility of extending PARPi efficacy to non BRCA1/2 mutated cancers through the employment of BETi [72]. Finally, the evidence that BETi treatment impairs DSB repair indicates that these drugs may also improve cytotoxic effects of DNA damaging agents like radiation or platinum-based chemotherapy.

### BRD4 and telomere regulation

Telomeres sense somatic cells aging by shortening at each mitosis and induce cell death when they become critically short. Thus, progressive reduction of telomere length is a rate limiting step for uncontrolled and unlimited cell proliferation capacity. Aberrant telomere elongation and alterations in Telomerase expression and/or activity are functional to cancer aggressiveness [6, 73]. Telomerase Reverse Transcriptase (TERT) is the catalytic subunit of Telomerase complex and together with its RNA template, the Telomerase RNA Component (TERC), are considered the limiting components of this complex [74]. Recently, independent reports highlighted a possible role of BRD4 in aberrant telomere regulation in cancer, even if the mechanisms through which BRD4 cooperates to telomere elongation is still poorly defined. First, BRD4 has been implicated in the aberrant transcriptional regulation of TERT, in TERT promoter mutated tumors. Recently, two recurrent mutations within the TERT promoter have been identified and associated with aggressiveness and poor prognosis in many types of cancer [75–79]. These mutations (− 124 bp C > T and -146 bp C > T upstream the TSS) represent the most prevalent non-coding alterations in cancer and create aberrant binding sites for E-Twenty-Six (ETS) TFs within the promoter, leading to increased TERT expression [77, 78]. Ankillar et al. using Circular Chromosome Conformation Capture (4C) and Chromatin Conformation Capture (3C), showed that the ETS factor GABPA binds specifically to mutant TERT promoter mediating long-range chromatin interaction and enrichment of active histone marks, driving TERT transcription. Furthermore the presence of GABPA at mutated promoter leads to an enrichment of active histone marks, and consequently drives TERT transcription [80]. According to these authors, BRD4 cooperates to this process through both a direct and an indirect mechanism. Directly BRD4 accumulates on TERT mutated promoter by binding to hyperacetylated histones and participates to the stabilization of the long-range chromatin interactions promoted by GABPA. Furthermore, BRD4 binds to GABPA promoter driving its transcription and fueling indirectly the transcriptional organization at the TERT mutated promoter resulting in increased TERT expression.

Treatment with BETi as well as BRD4 knock-down result in a significant reduction of TERT expression and impairment of Telomerase activity together with a significant decrease in the recruitment of histone active marks and long range chromatin interaction specifically in TERT promoter mutated cells. Consistently, CRISPR-mediated reversal of mutant TERT promoter or deletion of its long-range chromatin interaction, abrogates GABPA binding and long-range chromatin interactions, causing a loss of active histone marks and RNA-PolII recruitment with a consequent decrease of TERT transcription [80]. In addition to its function in promoting TERT expression, a non-transcriptional role of BRD4 in telomere homeostasis regulation has been proposed [81]. Using an unbiased screening based on a lentiviral shRNAs library targeting 706 known kinases and relative genes, BRD4 has been identified as necessary for telomere maintenance in cancer cells. Treatment with four different BRD4 pharmacological inhibitors, including OTX015, induced a dose-dependent telomere lengthening inhibition. Long-term BRD4 inhibition caused telomere shortening which was sufficient to cause telomere dysfunction and chromosomal fusion leading to cell death. Noticeably, this effect is not-dependent on Telomerase expression or enzymatic activity since BRD4 inhibition leads to telomere alterations even in experiments conducted in artificial cellular systems where TERT and TERC are exogenously overexpressed [81].

**Fig. 3:**
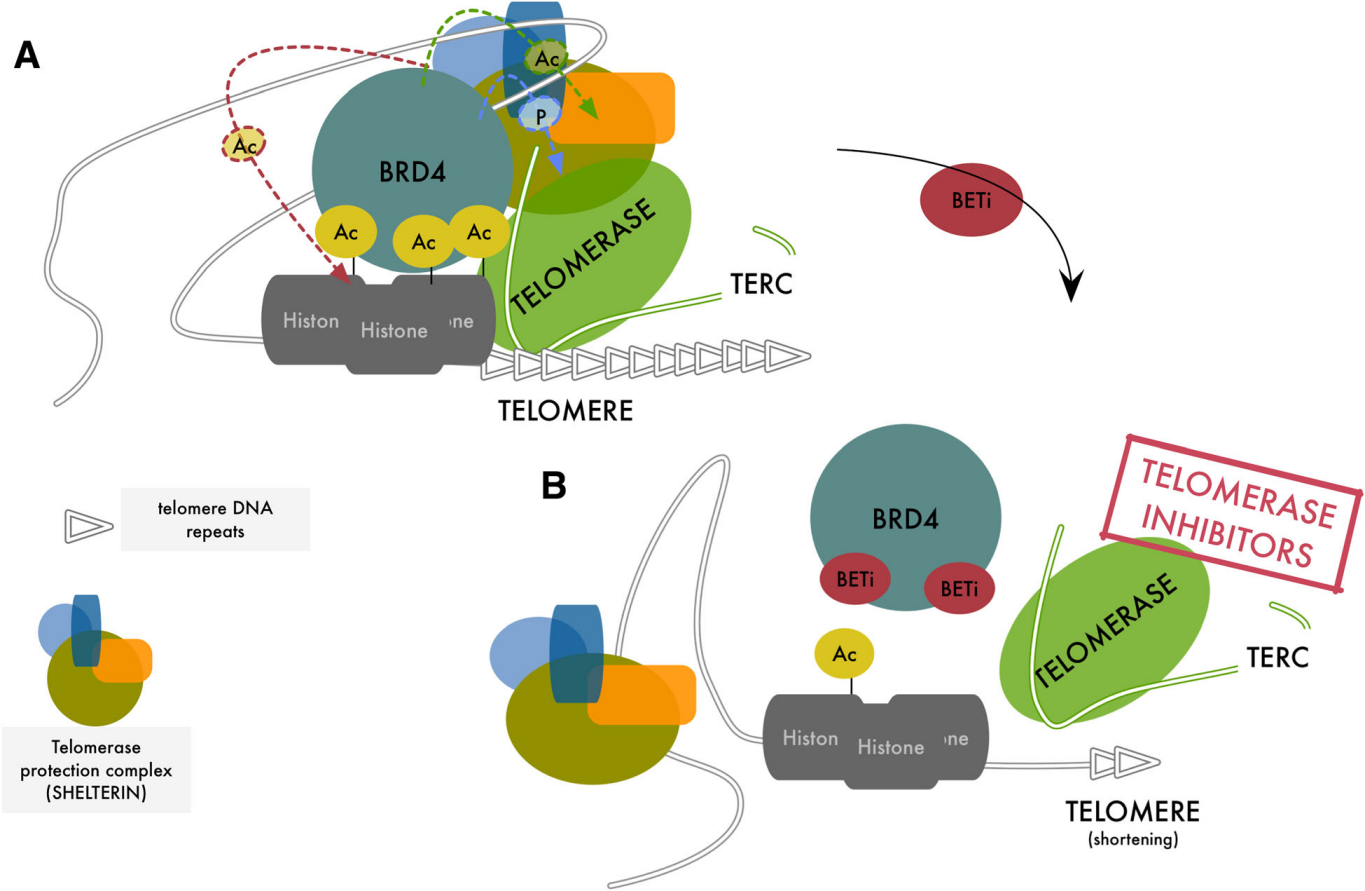
**a** Schematic representation of the possible BRD4 functions in telomere elongation. Increased histone acetylation and H2AX phosphorylation accumulate on telomeres, promoting BRD4 binding that in turn facilitates the assembly of telomere protection complex and promotes the activity of Telomerase. Since post-translational modification plays a fundamental role in telomere complexes regulation, it is likely that the kinase and/or the actetyltransferase function of BRD4 may take part to this process. For example, BRD4 may further promote acetylation of histone in the telomeric regions (red arrow) which in turn promotes telomere elongation. In addition, BRD4 may directly affect the acetylation (green arrow) or phosphorylation (blue arrow) of telomerase or other proteins of the telomere protection complex, promoting their activity. Indeed, TERT phosphorylation has been shown to be required for telomerase activation. **b** Effect of BETi on telomere regulation. BETi compete with acetylated histones for the binding at the BRD4 bromodomains releasing BRD4 from short telomere, destabilizing telomere protection complex organization and reducing Telomerase activity. The progressive shortening of telomere leads cancer cells to senescence or cell death. This structural effect in addition with the cancer specific effect of BRD4 on TERT promoter regulation may represent a possible strategy to target Telomerase function selectively in cancer cells or a strategy to improve telomerase inhibitors efficacy

How BRD4 orchestrates telomere maintenance is not fully defined. Short telomeres are characterized by increased histone acetylation and H2AX phosphorylation [82]. Since Telomerase complex preferentially acts on short telomere [83, 84], we may speculate that, BRD4 acts as a platform to recruit and stabilize the binding of the Telomerase complex and/or other telomere-associated complexes promoting telomeres lengthening by accumulating to hyper-acetylated histones at the end of chromosomes [83] (Fig. 3 a-b). Consistently, it has been demonstrated that treatment with HDAC inhibitors increases histone acetylation levels at telomere and enhances elongation also in absence of TERC (even if in minor extent), while HAT inhibition induces telomere shortening leading to telomere loss [85].

Targeting telomerase and telomere aberrant regulation has been long regarded as a promising anti-cancer strategy and Telomerase inhibitors have shown effectiveness in reducing cancer cell viability, tumor growth and metastasis in preclinical studies. However, these drugs lack sufficient cancer specificity and failed clinical trials due to high side effects toxicity [86–88]. The relevance of BRD4 in controlling TERT expression selectively in presence of cancer-associated promoter mutations, seems to suggest that inhibition of BRD4 by BETi could represent an alternative and more selective inhibition of telomerase. Besides, possible combination with BETi could improve effectiveness of telomerase inhibitors at lower doses, reducing the magnitude of undesired side effects.

## Conclusion

Accumulating evidence identifies BRD4 as master keeper of genome function and stability and underlines a common model of action across many processes in which BRD4 is involved. Reading the histone code, BRD4 senses hyper-acetylated chromatin sites (like enhancers, DSBs or telomeres) and accumulates on these loci favoring the recruitment and the stabilization of functionally relevant multiprotein complexes. The high-density protein binding changes chromatin physical status and creates separated functional domains optimizing proteins crosstalk and function.

The uprising evidence of the transversal centrality of BRD4 in many cancer supporting processes provides new interpretation for the cytotoxic activity of BETi and in particular envisages new and wide possibilities for the clinical employment of these drugs in cancer settings.

## Abbreviations

53BP1: p53 Binding Protein
AID: Activation Induced cytidine Deaminase
ATR: Ataxia Telangiectasia And Rad3-Related kinase
BD: Bromodomain
BETi: Bromodomain and Extraterminal inhibitor
BRCA1, BRCA2: Breast Related Cancer Antigens
BRD (1–4): Bromodomain-containing protein (1–4)
CCNE1: Cyclin E1
CCNT1: Cyclin T1
CDC6: Cell Division Cycle 6
CDK9: Cyclin-dependent kinase 9
CEBP: Ccaat-enhancer-binding proteins
CHD4: Chromodomain Helicase DNA Binding Protein 4
CHK1: Checkpoint kinase 1
CK2: Casein kinase 2
CTD: Carboxy-terminal domain
DDR: DNA damage repair
DSBs: DNA double strand breaks
ENH: Enhancer
ERG: ETS-related gene
eRNA: Enhancer RNA
ET: Extraterminal domain
ETS: E-Twenty-Six
FLI1: Friend leukemia integration 1 transcription factor
GABPA: GA-binding protein alpha
γH2AX: phosphorylated Histone 2AX
H3K27Ac: Histone 4 Lysine 27 Acetylated
H3K4me1: Histone 4 Lysine 3 monomethylated
H4Ac: Histone 4 acetylation
H4R3me1: Histone 4 Arginine 3 monomethylated
H4R3me2: Histone 4 Arginine 3 dimethylated
HAT: Histone acetyltransferase
IGLL5: Immunoglobulin lambda like polypeptide 5
IR: Ionizing radiation
IRF4: Interferon regulatory factor 4
JMJD6: Jumonji Domain-Containing Protein 6
MED1: Mediator-1
NELF: Negative Elongation Factor
NFKB: Nuclear factor kappa-light-chain-enhancer of activated B cells
NHEJ: Non Homologus End Joining
NSD3: Nuclear Receptor Binding SET Domain Protein 3
p300/CBP: p300/ CREB-binding protein
PARPi: Poli ADP-ribosio polimerasi inhibitors
PIC: PreInitation Complex
P-TEFb: Positive transcription elongation factor-b
RNA-PolII: RNA- Polimerase II
SE: Super-Enhancer
SMARCA4 SWI/SNF: Related, matrix associated, actin dependent regulator of chromatin, subfamily a, member 4
SMC2, SMC4: Structural Maintenance of Chromosomes 2 and 4
TEAD1: Transcriptional enhancer factor TEF-1
TERC: Telomerase RNA Component
TERT: Telomerase Reverse Transcriptase
TF: Transcription Factor
TSS: Transcription Starting Site
XBP1: X-box binding protein 1
